# Structural Basis for the Inverted Repeat Preferences of *mariner* Transposases*

**DOI:** 10.1074/jbc.M115.636704

**Published:** 2015-04-13

**Authors:** Maryia Trubitsyna, Heather Grey, Douglas R. Houston, David J. Finnegan, Julia M. Richardson

**Affiliations:** From the ‡ Institute of Cell Biology and; § Institute of Structural and Molecular Biology, School of Biological Sciences, University of Edinburgh, The King's Buildings, Max Born Crescent, Edinburgh EH9 3BF, Scotland, United Kingdom

**Keywords:** DNA recombination, DNA-protein interaction, molecular genetics, nucleic acid enzymology, phosphoryl transfer, structural biology, X-ray crystallography, DNA transposition

## Abstract

The inverted repeat (IR) sequences delimiting the left and right ends of many naturally active *mariner* DNA transposons are non-identical and have different affinities for their transposase. We have compared the preferences of two active mariner transposases, Mos1 and Mboumar-9, for their imperfect transposon IRs in each step of transposition: DNA binding, DNA cleavage, and DNA strand transfer. A 3.1 Å resolution crystal structure of the Mos1 paired-end complex containing the pre-cleaved left IR sequences reveals the molecular basis for the reduced affinity of the Mos1 transposase DNA-binding domain for the left IR as compared with the right IR. For both Mos1 and Mboumar-9, *in vitro* DNA transposition is most efficient when the preferred IR sequence is present at both transposon ends. We find that this is due to the higher efficiency of cleavage and strand transfer of the preferred transposon end. We show that the efficiency of Mboumar-9 transposition is improved almost 4-fold by changing the 3′ base of the preferred Mboumar-9 IR from guanine to adenine. This preference for adenine at the reactive 3′ end for both Mos1 and Mboumar-9 may be a general feature of *mariner* transposition.

## Teaser

**Background:** Transposases orchestrate movement of DNA transposons around and between genomes.

**Results:** Structural and biochemical approaches are combined to dissect the DNA preferences of two mariner transposases in each step of transposition.

**Conclusion:** Two active mariner transposases preferentially associate with and process one of their transposon ends.

**Significance:** The efficiency of mariner DNA transposition can be improved by optimizing transposon end sequences.

## Introduction

**FIGURE 1.**
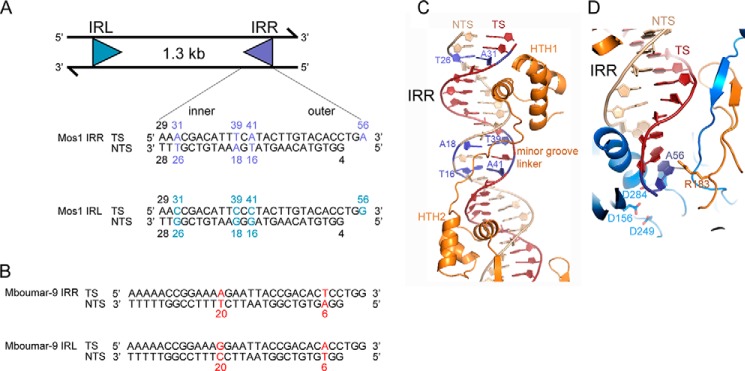
**Imperfect inverted repeats of two active *mariner* transposons, *Mos1* and *Mboumar-9*.** *A*, schematic of the Mos1 transposon and the sequences of the imperfect IRR and IRL. The TS and NTS sequences shown are the products of the staggered DNA cleavage reactions, which leaves a single-strand overhang of 3 bases on the 3′ end of the TS. The sequences of the Mos1 IRR and IRL differ at four positions, indicated in *purple* and *cyan*, respectively. *B*, sequences of the imperfect 32-bp inverted repeats of the *Mboumar-9* transposon. IRR and IRL of *Mboumar-9* transposon differ by two positions: 6 and 20, indicated in *red. C*, interactions between the Mos1 IRR and the Mos1 transposase DNA-binding domain in the Mos1 IRR PEC structure (PDB ID: 3HOS). The base pairs that differ between IRR and IRL are colored *purple. D*, interactions between the Mos1 IRR and the transposase catalytic domain.

DNA transposons of the *mariner/Tc1* family are useful genetic tools for manipulating eukaryotic genomes (1–3). They consist of DNA sequences 1–2 kb in length, with inverted repeat (IR)^2^ sequences (28–200 bp) at either end (see Fig. 1*A*). Cut-and-paste DNA transposition requires a transposase, often encoded within the transposon, which binds sequence-specifically to the transposon IRs, makes a staggered double-strand break at each IR, and then inserts the cleaved transposon ends (together with the gene they encompass) at a new genomic location. *mariner/Tc1* transposons usually integrate at TA dinucleotides, resulting in signature duplication of this sequence either side of the transposon.

For genetic manipulation applications, the DNA sequence between the inverted repeats can be replaced by a gene of interest; this can be as short as 100 bp or as large as a bacterial artificial chromosome (4). If transposase is supplied from another source, the gene can be cut out and moved to a new genomic location by transposition. The efficiency of such a system can be improved by optimizing the amino acid sequence of the transposase, for example by evolutionary reconstruction (5) (the strategy used to produce highly active Sleeping Beauty transposase (6)) or by random (7) or rational mutagenesis (8, 9), approaches taken to generate hyperactive Himar1 and Hsmar1 transposases. In the case of Tn5, further gains in DNA transposition efficiency were also achieved by changing the sequence of the IR DNA delimiting the transposon ends to optimize the interactions with hyperactive transposase (10).

The *mariner/Tc1* family of transposons is particularly widespread in nature (11–13). Many of the naturally active *mariner* transposons have imperfect IRs, containing DNA sequences that differ at each end, and transposase binds to these imperfect sequences with different affinities. The well characterized *mariner* transposon Mos1, found in *Drosophila mauritiana* (14), has 28-bp inverted repeats that differ by 4 bp (see Fig. 1*A*). This natural arrangement is suboptimal for transposition *in vitro* (15). The closely related *mariner* transposon Mboumar-9 from *Messor bouvieri* (16, 17) has 32-bp inverted repeats, which differ by 2 bp (see Fig. 1*B*).

Our crystal structure of the Mos1 paired-end complex (PEC), containing a Mos1 transposase dimer and two right IR (IRR) DNA duplexes, provided the first structural insight into transposase recognition of the Mos1 transposon ends (18, 19). The inner part of the IRR DNA sequence is recognized by the Mos1 DNA-binding domain of one transposase monomer, in *cis* (see Fig. 1*C*); this domain (residues 1–112) comprises two helix-turn-helix (HTH) motifs connected by a minor groove-binding linker. The outer IRR sequence (containing three unpaired bases at the reactive 3′ end that mimic the product of the staggered double-strand break) is recognized by the catalytic domain of the other transposase monomer, in a *trans* arrangement (see Fig. 1*D*).

Previously, it was shown that the N-terminal 150 residues of Mos1 transposase, containing the DNA-binding domain, have 5–10 times higher affinity for the right Mos1 IR sequence than the left IR (IRL) (20, 21). A similar result was observed for a full-length MBP-Mos1 transposase fusion (15). The difference in affinity for the two ends was attributed primarily to the base difference at position 16 (15, 22), in the region bound by the minor groove-binding linker in the Mos1 PEC crystal structure (see Fig. 1*A*).

To understand the structural basis of this reduced binding affinity, we have determined a crystal structure of the Mos1 PEC containing the IRL transposon sequence, to a resolution of 3.1 Å. This reveals subtle differences in the interactions of the minor groove-binding linker with the IRL sequence and explains the reduced affinity of the Mos1 DNA-binding domain for this end. Surprisingly, the structure also reveals additional interactions between the guanine base at the reactive 3′ end of the IRL and Glu-345, the C-terminal amino acid of Mos1 transposase, which likely restrain the position of 3′ base and hinder transposition.

We also compared the IR preferences in the subsequent steps of the transposition reaction: DNA cleavage and DNA strand transfer. To establish whether there are common features in the recognition and activity of *mariner* transposon ends, we compared the activities of Mos1 and Mboumar-9 transposases. We found that both transposases have a preferred end for *in vitro* cleavage and *in vitro* strand transfer. Moreover, we found that an adenine base at the reactive 3′ transposon end is optimal for both Mos1 and Mboumar-9 *in vitro* transposition. On this basis, we improved the efficiency of Mboumar-9 transposition by 3.9-fold.

## Experimental Procedures

### 

#### 

##### Construction of Donor Transposon Plasmids

Plasmids containing a kanamycin resistance (kanR) gene flanked by two transposon IRL sequences, two IRR sequences, or one IRL and one IRR were created by first amplifying the kanR cassette from pUC4K with two primers carrying the Mos1 IRR or IRL sequences or the Mboumar-9 IRR or IRL sequences. Additionally, the primers introduced either a SacI site (with IRL) or an XbaI site (with IRR). This enabled cloning of the 1.3-kb PCR products into the pEP185.2 plasmid containing the conditional origin of replication, oriR6K. In this way six donor transposon plasmids (pEPMosLL, pEPMosLR, pEPMosRR, pEPMboLL, pEPMboLR, and pEPMboRR) were generated.

Site-directed mutagenesis was performed on the pEPMboLL plasmid to create a new donor transposon (pEPMboLL-G3′A) in which the guanine base at the 3′ end of both IRLs was replaced by adenine. DNA sequencing confirmed the presence of the mutations.

##### Mos1 and Mboumar-9 Transposases

Both transposases contained the mutation T216A, previously shown to promote expression of soluble Mos1 in *Escherichia coli*, and were purified as described previously (17, 23). Mos1 transposase was stored at −20 °C in 50% (v/v) glycerol, 25 mm Tris, pH 7.5, 250 mm NaCl, 1 mm DTT. Mboumar-9 transposase (0.5 mg/ml, 82% pure) was snap-frozen in 20 mm PIPES, pH 6.8, 500 mm NaCl, and 1 mm DTT and stored at −80 °C.

##### Preparation of DNA Substrates

Duplex IRL and IRR DNA substrates were prepared by annealing the 28-nt transferred strand (TS) with its complementary 25-nt non-transferred strand (NTS) in TEN buffer (10 mm Tris, pH 8.0, 100 mm NaCl, 1 mm EDTA). These oligonucleotides had the sequences shown in Fig. 1*A* and were synthesized by Integrated DNA Technologies. For the strand transfer assays, the fluorescent label IRDye® 700 was incorporated at the 5′ end of the TS (oligonucleotides synthesized by Metabion) to enable detection. The 50-mer target DNA substrate, containing one TA dinucleotide, was prepared by annealing the sequence 5′-AGCAGTCCACTAGTGCACGACCGTTCAAAGCTTCGGAACGGGACACTGTT with its complementary strand, followed by PAGE purification.

##### Preparation and Crystallization of the Mos1 IRL PEC

Analogous to preparation of the Mos1 PEC containing the IRR (19), full-length transposase was mixed with duplex IRL DNA (500 μm) in a 1:1.5 protein:DNA molar ratio in 25 mm Tris, pH 7.5, 250 mm KCl, and 1 mm DTT. The final PEC concentration was 52 μm. Crystals were grown by vapor diffusion in hanging drops in Linbro plates at 17 °C. The well solution contained 100 mm sodium citrate, pH 5.8, 100 mm ammonium acetate, 450 mm KCl, and 5 mm MnCl_2_. To improve diffraction quality, initial crystals were used for micro-seeding. A seed stock was prepared by diluting three crushed crystals in 100 μl of well solution. Crystals were grown in hanging drops containing 1.5 μl of complex, 1.5 μl of well solution, and 1.5 μl of a seed stock and flash-frozen prior to data collection, as described previously for the Mos1 IRR PEC (19).

##### X-ray Data Collection and Structure Refinement

X-ray diffraction data were collected at the European Synchrotron Radiation Facility (ESRF) (BM14) equipped with an ADSC quantum 210 detector. The crystal displayed monoclinic P2_1_ symmetry and diffracted x-rays to a maximum resolution of 3.1 Å. Initial phases were determined by molecular replacement using the structure of the Mos1 PEC containing IRR DNA (Protein Data Bank (PDB) ID: 3HOS) as the model. Coot (24) was used to view the maps and manipulate the structure. Restrained refinement was performed with Refmac in the CCP4 suite (25) and included weak non-crystallographic symmetry restraints on the protein atoms. The refinement statistics are shown in Table 1. The Ramachandran statistics were calculated using MOLPROBITY. All structural figures were prepared using PyMOL.

##### Molecular Docking

The IRR and IRL DNA structures were docked onto the protein from the IRL PEC structure using AutoDock 4.2.3 (26). To prepare the required input files, water molecules and other heteroatoms were first removed from the crystal structures. Then position-optimized hydrogen atoms were assigned using the program PDB2PQR 1.6 (27), utilizing the optional PropKa algorithm (28) with a pH of 7.4 to predict protonation states. The AutoDock Tools 1.5.4 utilities prepare_receptor4.py and prepare_ligand4.py were used to assign Gasteiger charges to protein atoms and Gasteiger charges and hydrogen atoms to the DNA ligands, respectively.

The size of the docking search space was set at 4 Å around the DNA ligands (*i.e.* 8 Å was added to each of the maximum *x*, *y*, and *z* dimensions of the molecules), with the center of the ligand defining the center of the search space. The AutoGrid grid point spacing was set at 0.2 Å. The AutoDock parameter file specified 10 Lamarckian genetic algorithm runs for each docking with 5,000,000 energy evaluations and random initial placement (translation and rotation) of the ligand.

##### In Vitro Plasmid Cleavage Assay

Donor transposon plasmid (5.6 kb, 500 ng, 7.24 nm) was incubated with Mos1 or Mboumar-9 transposase (protein:DNA molar ratio 5:1) in 20-μl final volume for 1 h at 30 °C in a buffer containing: 25 mm HEPES, pH 7.5, 12.5 μg/ml BSA, 2 mm DTT, 100 mm NaCl, 10% (v/v) glycerol, and 10 mm MnCl_2_ or MgCl_2_. To stop the reaction, 0.4 μl of 500 mm EDTA was added, and the products were analyzed by agarose (1% w/v) gel electrophoresis. The percentage of backbone released was calculated by comparing the intensity of the backbone bands with the total intensity in the lane averaged from four gels.

##### In Vitro Transposition Assay

Transposon donor plasmid (5.6 kb, 500 ng, 7.24 nm) was incubated with the pBSKS+ target plasmid (3 kb, 300 ng) and 72.4 nm transposase for 1 h at 30 °C in 20-μl final volume. The buffer contained 25 mm HEPES, pH 7.5, 100 mm NaCl, 10% (v/v) glycerol, 2 mm DTT, 200 μg/ml acetylated BSA, and 10 mm MnCl_2_ or MgCl_2_. The reaction was stopped by the addition of 80 μl of buffer containing 50 mm Tris, pH 7.5, 500 μg/ml proteinase K, 10 mm EDTA, and 6.25 μg/ml yeast tRNA and incubated for 1 h at 37 °C. DNA was phenol-extracted, ethanol-precipitated, and resuspended in 10 μl of nuclease free water (Qiagen). Chemically competent cells (100 μl) *E. coli* DH10B (10^7^ CFU/μg) were transfected with 10 μl of DNA, and after heat shock and recovery, half was plated out on LB agar containing kanamycin (50 μg/ml) to select for transposition products. Up to 8,000 colonies per 1 μg of the donor plasmid were obtained, corresponding to a transposition efficiency of 8 × 10^−4^.

##### Transposon Strand Transfer Assay

Reactions contained 15 nm 50-mer target DNA, 1.5 nm IRR or IRL DNA and 15 nm Mos1 or Mboumar-9 transposase in a final volume of 20 μl containing 25 mm HEPES, pH 7.5, 50 mm Potassium Acetate, 10% (v/v) glycerol, 0.25 mm EDTA, 1 mm DTT, 10 mm MgCl_2_, 50 μg/ml BSA and 20% (v/v) dimethyl sulfoxide (DMSO). Reactions were incubated for 2 h at 30 °C, and the products were separated on an 8% denaturing polyacrylamide gel as described previously (18, 29). To visualize the products, the IRDye® 700 was excited at 680 nm and detected on a LI-COR Odyssey CLx system. The fluorescence intensities of the product bands were quantified using Image Studio software. The percentage of integration is calculated by dividing the intensity of the product band with the total fluorescence intensity in the lane.

## Results

### 

#### 

##### The Imperfect Inverted Repeat Sequences of Mos1 and Mboumar-9

The 28-bp right and left IRs of Mos1 differ at four positions (Fig. 1*A*). Each of these involves substitution of A or T in the IRR for G or C in the IRL. Consequently, the IRR sequence has a higher AT content (64.3%) than the IRL sequence (50%). Three of the substitutions are located in the inner IR, at positions 16, 18, and 26 of the NTS. In the Mos1 IRR PEC crystal structure, T26 and its complementary base (A31 on the TS) are close to the major groove recognized by the first HTH motif (Fig. 1*C*). Both T16 and A18, and their complementary bases (A41 and T39, respectively), are in the region recognized by the minor groove-binding linker (Fig. 1*C*). None of the bases differing between the IRR and IRL are involved in direct base to side-chain interactions with the Mos1 transposase DNA-binding domain. The fourth difference between the IRR and IRL sequences is at the reactive 3′ end of the transposon IR, at position 56. In the Mos1 IRR PEC crystal structure, A56 is unpaired and makes a purine-specific contact with the side chain of Arg-183, via N7 (Fig. 1*D*).

The 32-bp Mboumar-9 IRL and IRR sequences differ at only two positions: 6 and 20 of the non-transferred strand (Fig. 1*B*). The base at position 6 is A in the IRR sequence and T in the IRL sequence, whereas T20 in the IRR is C20 in the IRL sequence. The base pairs at the equivalent positions of the Mos1 IRR have no contacts with the protein in the Mos1 PEC structure.

##### Crystal Structure of the Mos1 PEC with the Transposon IRL DNA Sequence

Crystals of the Mos1 IRL PEC were formed by mixing full-length Mos1 transposase (T216A mutant) with duplex IRL DNA (Fig. 1*A*). This had a 3-nt overhang at the 3′ end of the transferred strand, mimicking the product of staggered transposon excision. The monoclinic crystals diffracted x-rays to a maximum resolution of 3.1 Å. The crystallographic phases were determined by molecular replacement using our previous structure of the Mos1 IRR PEC as the model (PDB ID: 3HOS). The data collection, merging, and refinement statistics are shown in Table 1.

**TABLE 1:**
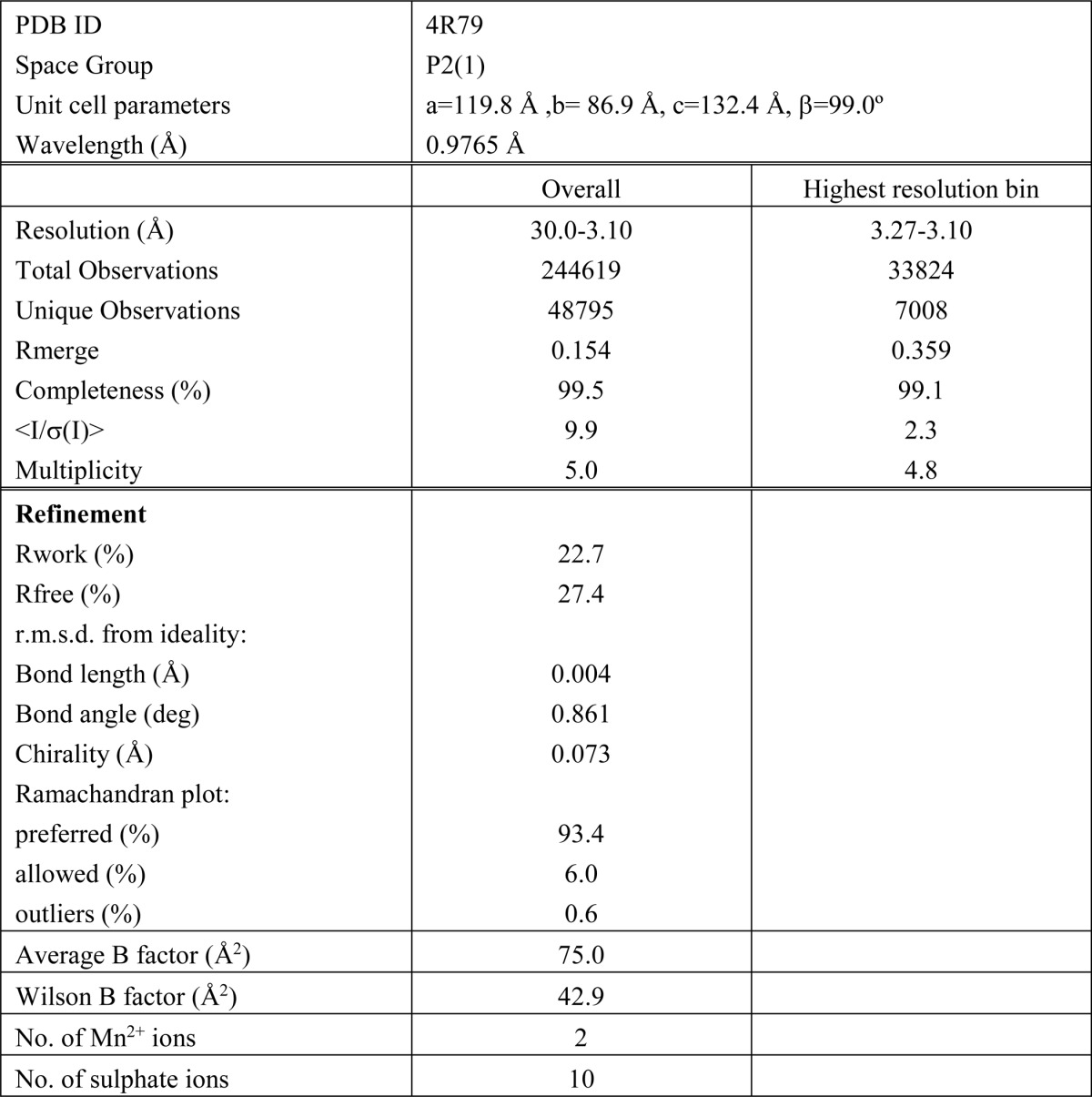
**X-ray diffraction data and refinement statistics** r.m.s.d., root mean square deviation.

The Mos1 IRL PEC structure has a similar overall architecture to the Mos1 IRR PEC described previously (18) and comprises a transposase dimer bound to two IRL DNA molecules in a crossed configuration; one IRL DNA is bound by the DNA-binding domain of one monomer and the catalytic domain of the other monomer, and vice versa. As before, two additional IRL DNA duplexes interact with the catalytic domains, possibly occupying the binding sites of target DNA (30).

##### Comparison of Transposase-DNA Interactions in the Mos1 IRL and IRR PECs

**FIGURE 2.**
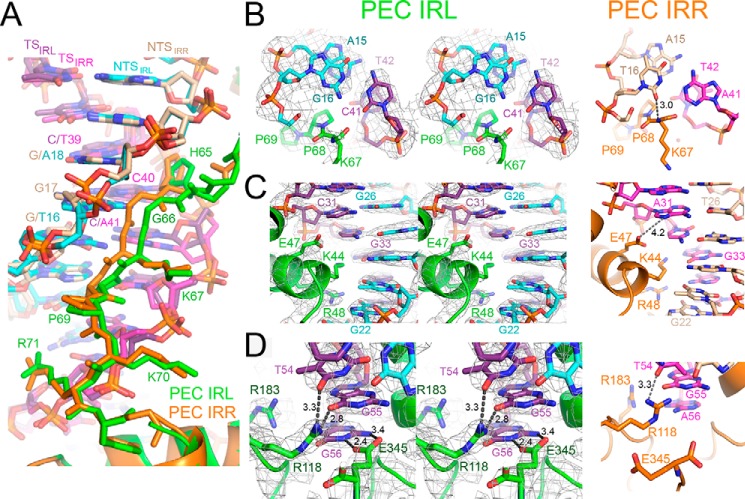
**Transposase-DNA interactions in the crystal structure of the Mos1 IRL PEC and comparison with the IRR PEC structure.** *A*, transposase residues His-65 to Arg-71, linking the HTH1 and HTH2 motifs, bind in the minor groove between bases 15–18 of the NTS. The linker is displaced out of the minor groove in the PEC IRL (*green*) as compared with the equivalent residues in the PEC IRR (*orange*). *B*, *cis* linker-DNA interactions involving the base pair G16:C41 in the PEC IRL (*left panel*). A stereo view is shown, and the 2*F_o_* − *F_c_* electron density map, contoured at 2σ, is displayed as a *gray mesh*. The *right panel* shows the equivalent interactions in the PEC IRR involving the T16:A41 base pair. *C*, interactions between HTH1 and the major groove in the inner region of the inverted repeat sequence. *D*, *trans* interactions between the catalytic domain and DNA involving the unpaired base G56 in the IRL PEC and A56 in the IRR PEC.

The linker between HTH1 and HTH2 binds in the minor groove of IR DNA between nucleotides 15 and 18 on the non-transferred strand primarily by shape complementarity (Figs. 1*C* and 2*A*). The lower affinity of transposase for the IRL has been attributed mainly to sequence differences in this region (15). In the Mos1 IRL PEC, the linker is displaced out of the minor groove, by a maximum of 1.7 Å at the amide bond between His-65 and Gly-66, as compared with its position in the IRR PEC (Fig. 2*A*). This is due in part to the higher GC content of the IRL sequence in this region as compared with the IRR sequence; the guanine 2-amino group adds bulk and hydrogen bond donors in the minor groove that obstruct transposase linker binding. Moreover, the pyrimidine-specific interaction between T16 O_2_ and the backbone amide of Lys-67 in the IRR PEC is lost in the IRL PEC because T16 is replaced by G in the IRL sequence (Fig. 2*B*). Additionally, the O_2_ of C41 (base-paired with G16) repels the backbone carbonyl of Lys-67 in the PEC IRL structure.

Other features of this central IR region are similar in both the IRR and IRL PEC structures. The pyrimidine-specific interaction between O_2_ of C40 and HD1 of the His-65 side chain is maintained, and there are no changes in the minor groove width between the two structures, as has been observed for example in interactions of the Fis-binding protein with minor groove DNA (31). This is similar to an AT hook (palindromic consensus sequence Pro-Arg-Gly-Arg-Pro), which inserts into AT-rich minor grooves so that the Arg side chains run parallel with the groove, without bending, expanding, or unwinding the double helix (32).

HTH1 binds in the major groove of the inner IR in both the IRL and IRR PEC structures; the side chains of Arg-48 and Lys-44 form base-specific interactions with G22 and G33, respectively (Fig. 2*C*). In this region, the proximity of the carboxylate side chain of Glu-47 and the N7 of A31 in the IRR sequence (4.2 Å apart) provides the potential for an additional, purine-specific interaction, mediated either by a water molecule or by protonation of the Glu-47 side-chain carboxylate. However, no such additional interaction would be possible with the IRL sequence, where A31 is replaced by C31.

At the reactive 3′ end of the inverted repeat, the three unpaired bases on the transferred strand are held in place by sequence-specific interactions with the transposase clamp loop residues. Arg-118 contacts both T54 and G55, and Arg-183 interacts with the N7 of the terminal purine; this is A56 in the IRR and G56 in the IRL sequence (Fig. 2*D*). These interactions position the terminal 3′-OH (the nucleophile for the strand transfer reaction) in close proximity to the active site. Surprisingly, in the IRL PEC structure, there are additional interactions between the N_1_H and NH_2_ of G56 and the side-chain carboxylates of Glu-345, the C-terminal transposase residue.

It should be noted that the 3′ end of the transferred strand becomes unpaired only after the complementary non-transferred strand has been cleaved and removed from the active site. Thus, the extra hydrogen bonds between Glu-345 and the 3′-G would contribute to the stability of the IRL PEC only *after* first strand cleavage. The higher binding affinity of the transposase for the un-cleaved IRR as compared with the un-cleaved IRL, as observed in previous gel retardation experiments (20), is consistent with the differences in the minor groove linker interactions in the IRR and IRL PEC structures seen here.

The free energy of binding (including van der Waals, H-bond, and electrostatic contributions) of the IR DNA and the protein was estimated via molecular docking. The IRL PEC was predicted to be 2.1 kcal/mol more stable than the IRR PEC. The free energy of an H-bond in which an amine group donates to a carbonyl oxygen in a water environment has been estimated to be between 0.5 and 1.6 kcal/mol (33). Thus, the docking results are consistent with the hypothesis that the additional stability of the IRL PEC can be accounted for by the addition of two hydrogen bonds between Glu-345 and G56 (the unpaired base at the 3′ end of the TS) and the loss of a hydrogen bond between T16 and the amide of Lys-67 in the linker region.

##### One of the Inverted Repeats Is Preferred for in Vitro Cleavage

**FIGURE 3.**
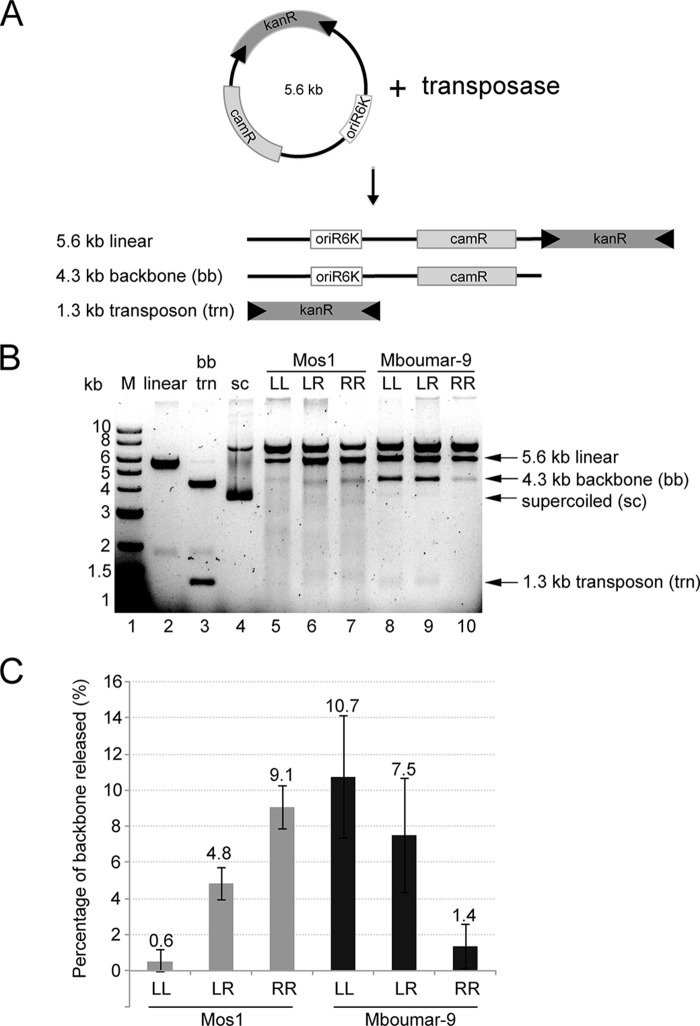
**Inverted repeat preferences of Mos1 and Mboumar-9 transposases for *in vitro* DNA cleavage of transposon-containing plasmids.** *A*, schematic of the *in vitro* plasmid DNA cleavage assay and the expected products. The donor DNA plasmid contains a transposon comprising a kanamycin resistance gene (kanR) flanked by inverted repeats (*black arrows*), a chloramphenicol resistance gene (camR), and the oriR6K origin of replication. *B*, agarose gel of the products of *in vitro* IR DNA cleavage. *Lane 1*, 1-kb DNA ladder of markers (*M*); *lane 2*, pEPMosLL linearized with XbaI; *lane 3*, pEPMboLL digested with SacI to excise the transposon; *lane 4*, supercoiled (*sc*) plasmid. *Lanes 5–7*, cleavage of the Mos1 transposon containing two left IRs (*LL*), one left and one right IR (*LR*), or two right IRs (*RR*). *Lanes 8–10*, cleavage of Mboumar-9 transposons. *C*, quantification of the percentage of plasmid backbone released (as a proportion of the total intensity of the lane) in each of the reactions in *lanes 5–10* above. The *error bars* indicate the S.D. between 4 independent measurements.

To establish whether there is an optimal arrangement of IRs for transposon excision, we performed *in vitro* cleavage reactions (20) (Fig. 3*A*). Transposons containing either the natural combination of IRR and IRL or two copies of IRR or IRL were created for both Mos1 and Mboumar-9. For Mos1, we found the highest level of transposon excision (9.1%) from the donor plasmid backbone using a transposon containing two Mos1 IRRs (Fig. 3, *B* and *C*). Mboumar-9 also had a preference toward one of the inverted repeats, in this case IRL with 10.7% excision (Fig. 3, *B* and *C*). In the most active excision reactions (Mos1 with IRR and Mboumar-9 with IRL), excised transposon bands of 1.3 kb can be observed.

The 15-fold higher cleavage activity of the Mos1 transposon with two IRRs as compared with the transposons containing two IRLs (Fig. 3*C*) is consistent with the 5–10 times higher binding affinity of the transposase DNA-binding domain for IRR as compared with IRL observed previously (15, 20). We also noted that there is more smearing from DNA on the lanes with Mos1 *in vitro* cleavage reactions (*lanes 5–7*), implying that Mos1 transposase may either contain contaminating nuclease or have stronger nonspecific nuclease activity than Mboumar-9 transposase.

##### Is There a Preferred IR Sequence for Strand Transfer?

After IR DNA excision, the TS reactive 3′-OH is poised for integration into target DNA. Transposons of the *mariner*/*Tc1* family usually integrate at a TA dinucleotide, and the transferred strand 3′-OH performs nucleophilic attack on the target DNA phosphate backbone at the 5′ side of the TA.

**FIGURE 4.**
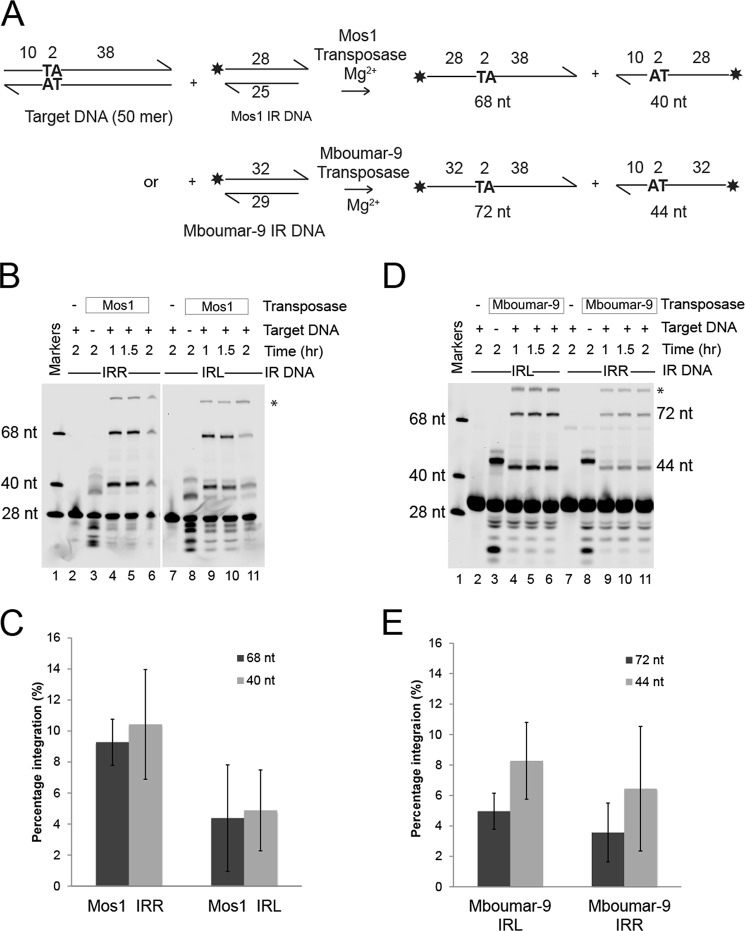
**Comparison of *in vitro* strand transfer of the left and right Mos1 and Mboumar-9 inverted repeats.** *A*, schematic of the assays. The *asterisk* indicates the position of the fluorescent label. The size of the products of strand transfer depends on which strand of the target DNA has been attacked. *B*, denaturing polyacrylamide gel of the Mos1 IR DNA strand transfer products. *Lane 1* contains fluorescent labeled markers; *lanes 2–6* contain labeled Mos1 IRR DNA; and *lanes 7–11* contain labeled Mos1 IRL DNA. *Lanes 2* and *7*, reactions without transposase; *lanes 3* and *8*, reactions with no target DNA. The products in *lanes 3* and *8* result from integration of IR DNA into the two TA dinucleotides contained within the Mos1 IR DNA sequence. The bands marked by the *asterisk* are most likely a result of the subsequent integration of the most prominent 37-nt product into target DNA. *C*, quantification of the percentage of total Mos1 strand transfer products (68 and 40 nt) after a 1.5-h incubation. *D*, denaturing polyacrylamide gel of the Mboumar-9 IR DNA strand transfer products; lane contents are as described in *B* except that Mboumar-9 transposase, IRR, and IRL DNA were used. The Mboumar-9 IR DNA sequences contain one TA nucleotide into which other IR DNA molecules can integrate, generating 47- and 51-nt products (*lanes 3* and *8*). Integration of the most abundant 47-nt product into target DNA would generate an 87-nt strand (marked by *asterisk*). *E*, quantification of the percentage of total Mboumar-9 strand transfer products (72 and 44 nt) after a 1.5-h incubation. *Error bars* in *panels C* and *E* indicate the S.D. between 2 and 3 experiments, respectively.

To establish whether there is a preference for either the IRR or IRL sequence in the strand transfer step of the transposition reaction, we performed *in vitro* strand transfer assays using “pre-cleaved” Mos1 IRR or IRL DNA substrates, incorporating a fluorophore at the 5′ end of the 28-nt transferred strand for detection of 68- and 40-nt strand transfer products, as shown schematically in (Fig. 4*A*). We also performed the assay with Mboumar-9 IRR and IRL DNA substrates and Mboumar-9 transposase; in this case, strand transfer yields labeled products of 72 and 44 nt (Fig. 4*A*).

We found that there was a preference for the Mos1 IRR in the strand transfer reaction, as the IRL showed 47% of the activity of the IRR (Fig. 4, *B* and *C*). However, the small preference for strand transfer of Mboumar-9 IRL as compared with Mboumar-9 IRR (which showed 76% of the strand transfer of the IRL) was within the error of the experiment (Fig. 4, *D* and *E*).

##### In Vitro Transposition of Natural Transposons Is Not the Most Efficient

**FIGURE 5.**
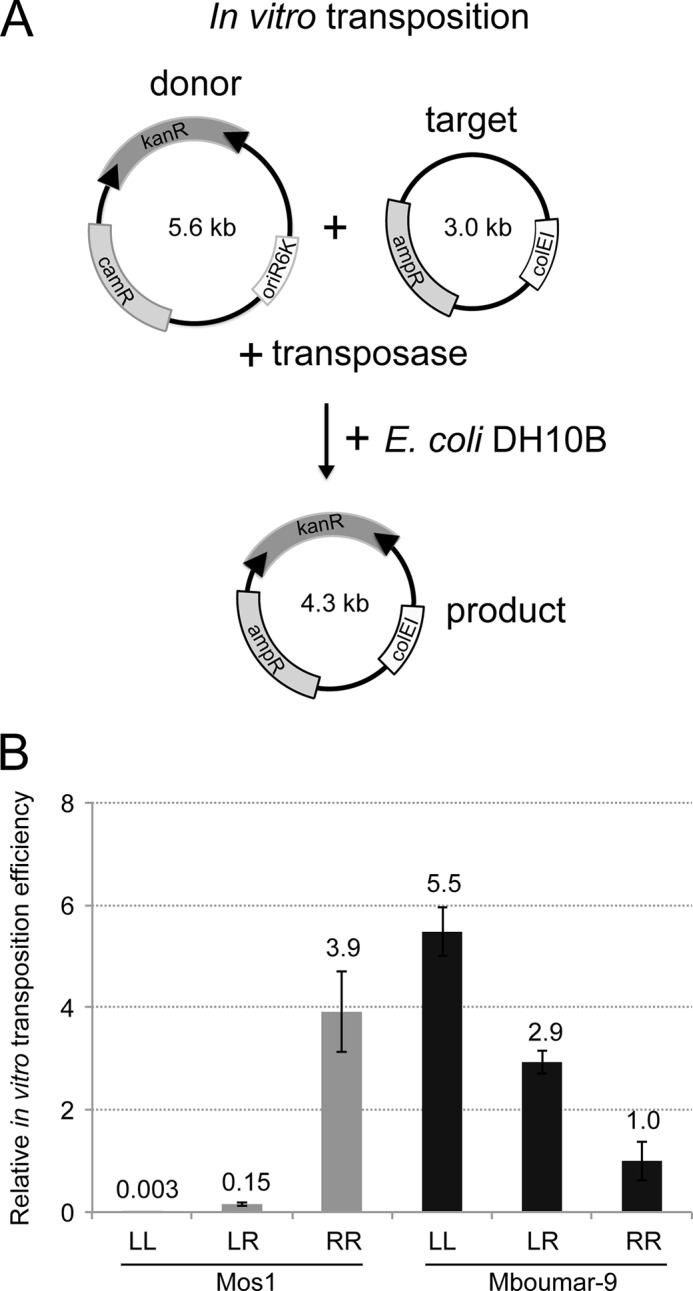
***In vitro* transposition efficiency of Mos1 and Mboumar-9 transposons flanked with different IRR and IRL combinations.** *A*, schematic of the *in vitro* transposition assay. The donor plasmid is incubated with a target plasmid, containing an ampicillin resistance (ampR) gene and a colE1 origin of replication, and purified transposase. The donor plasmid has a conditional origin of replication (oriR6K) and is unable to replicate in the recipient strain *E. coli* DH10B. The products of transposition are scored by counting the number of colonies carrying the kanR marker. The transposition efficiency was calculated as the number of kanR colonies per 1 μg of the donor plasmid divided by the transformation efficiency (CFU/μg). *B*, relative *in vitro* transposition efficiencies of Mos1 and Mboumar-9 donor plasmids containing different combinations of left and right IRs (as in Fig. 3*B*). These experiments were performed using MgCl_2_. For ease of comparison, the *in vitro* transposition efficiency was normalized to 1.4 × 10^−4^, the transposition efficiency of the Mboumar-9 pEPMboRR donor plasmid. Each experiment was conducted 4 times, and the *error bars* indicate the S.D. between measurements. *LL*, two left IRs; *LR*, one left and one right IR; *RR*, two right IRs.

Next we compared the *in vitro* transposition activity of Mos1 and Mboumar-9 transposons with all combinations of IRR and IRL, as shown schematically in Fig. 5*A*. As observed previously (15), the Mos1 transposon with the natural arrangement of one IRL and one IRR is less active than a transposon flanked with two copies of IRR (by a factor of 26). Furthermore, the transposon flanked by two IRLs is 50 times less active than the natural combination (Fig. 5*B*). We observed a similar result for Mboumar-9 *in vitro* transposition; the natural Mboumar-9 transposon, with imperfect IRs, is two times less active than a transposon with two copies of IRL (Fig. 5*B*) and three times more active than a transposon with two right inverted repeats. Thus, both Mos1 and Mboumar-9 show a preference for one end, and *in vitro* transposition is most efficient with a transposon containing two identical preferred ends. A similar result has also been observed for the Tc1 transposon Sleeping Beauty (34).

##### Replacing the 3′-Guanine of Mboumar-9 IR with a 3′-Adenine Increases the Rate of Mboumar-9 in Vitro Transposition Almost 4-fold

In the Mos1 IRR PEC, we observed a purine-specific interaction between Arg-183 and the N7 of the terminal 3′-A. This is preserved in the Mos1 IRL PEC structure, where the equivalent 3′ base is G. However, there are additional interactions between this 3′-G and the Glu-345 side-chain carboxylates that may contribute to the reduced cleavage and strand transfer activity of the IRL as compared with IRR, for example by restraining the position of the 3′ end of the IRL transferred strand too rigidly.

Both Mboumar-9 IRL and IRR sequences (16) have a G at the equivalent position at the 3′ end of the transferred strand (Fig. 1*A*). Moreover, Arg-183 and Glu-345 are conserved in the Mboumar-9 transposase. We therefore predicted that, as seen in the Mos1 IRL PEC, a purine-specific interaction can form between Mboumar-9 Arg-183 and the 3′-G, and that Mboumar-9 Glu-345 can make base-specific contacts with the 3′-G to down-regulate transposition.

**FIGURE 6.**
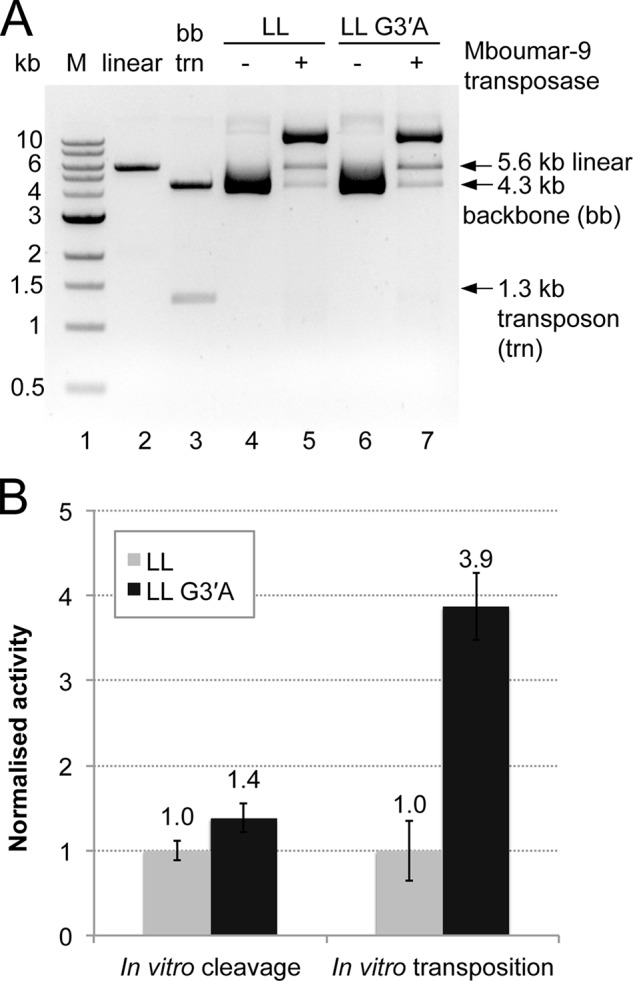
**Comparison of *in vitro* DNA cleavage and DNA transposition of the donor *Mboumar-9* transposons pEPMboLL (with two native IRLs) and pEPMboLL-G3′A (with two mutated IRLs).** *A*, agarose gel of the products of *in vitro* DNA cleavage. Experiments were performed twice. *Lane 1*, 1-kb DNA ladder of markers (*M*); *lane 2*, pEPMosLL linearized with XbaI; *lane 3*, pEPMboLL digested with SacI to excise the transposon; *lane 4*, pEPMboLL plasmid; *lane 5*, pEPMboLL plasmid incubated with Mboumar-9 transposase; *lane 6*, pEPMboLL-G3′A plasmid; *lane 7*, pEPMboLL-G3′A plasmid incubated with Mboumar-9 transposase. *LL*, two left IRs. *B*, quantification of *in vitro* cleavage and transposition of these transposons. Three repeats of the transposition reactions were performed. The percentage of backbone DNA released from the LL and LL-G3′A donor plasmids, as well as the relative efficiency of Mboumar-9 *in vitro* transposition, is normalized to that of LL. The *error bars* indicate S.D. between multiple measurements.

We asked whether swapping the 3′-G of the Mboumar-9 IRL with a 3′-A would change the efficiency of *Mboumar-9* transposition. To test this, we created a transposon donor plasmid, pEPMboLL-G3′A, in which the kanR sequence is flanked by two *Mboumar-9* IRL sequences with A replacing G at the 3′ end of the TS. First, we tested whether there is preference for excision of the pEPMboLL-G3′A transposon as compared with the plasmid containing two native IRLs: pEPMboLL. The results of the *in vitro* cleavage assay (Fig. 6*A*) showed that the pEPMboLL-G3′A mutant transposon is cleaved 1.4 times more efficiently from the donor backbone than the pEPMboLL transposon (Fig. 6*B*). A similar result was found for cleavage of the *Hsmar1* transposon (35). Next we compared the efficiency of the *in vitro* transposition reaction using the pEPMboLL or pEPMboLL-G3′A donor plasmids. Strikingly, there was a 3.9-fold enhancement in the efficiency of *in vitro* transposition with the mutant pEPMboLL-G3′A donor plasmid, as compared with pEPMboLL (Fig. 6*B*). This result suggests that the nature of the base at the 3′ end of the inverted repeat is a significant determinant of transposition efficiency.

## Discussion

Transposition of DNA transposons requires four steps: binding of transposase to the terminal IR, cleavage of both strands of DNA at each end of the transposon, strand transfer to link the 3′ ends of the excised transposon to target DNA, and sealing of the gap between the transposon 5′ ends and target DNA by DNA repair enzymes. The efficiency of transposition will be a product of the efficiency of each of these steps. We have identified factors that determine the efficiency of each of the first three steps, and we have investigated how they may be explained by structures of transposase bound to IR DNA.

The cut-and-paste *mariner* DNA transposon Mos1 occurs naturally with imperfect terminal IRs, and the Mos1 transposase has higher binding affinity for IRR than for IRL (20). Comparison of the crystal structure of the Mos1 IRL PEC reported here with our previous IRR PEC structure (18) provides a molecular explanation for this difference in binding affinity, as subtle differences in the interactions of the central region of IR and the linker between the two HTH regions of transposase are revealed.

We further dissected the effect of the imperfect IR sequences of Mos1 by comparing DNA cleavage and strand transfer at IRR and IRL. Our results show that both DNA cleavage and strand transfer are more efficient with the preferred Mos1 IRR sequence. These two effects combined give rise to the 26-fold higher efficiency of *in vitro* transposition of a Mos1 transposon with two IRRs as compared with the natural arrangement.

The dinucleotide CpA is found at the 3′ termini of many LTR retrotransposons, virtually all retroviral cDNAs, and some bacterial DNA transposons, *e.g.* Mu (36). Our results have shown that the nature of the base at the reactive 3′ transposon end is also important for *mariner* transposition efficiency. Our Mos1 PEC crystal structures show that the transposase makes purine-specific interactions with the 3′ base, consistent with DNase I footprinting data (21) The extra interactions observed between the 3′-G of the Mos1 IRL and the transposase C-terminal residue Glu-345 in the IRL PEC structure likely reduce DNA cleavage and strand transfer activity at this end by restraining the position of the TS 3′ end.

The closely related *mariner* transposon *Mboumar-9* also occurs naturally with imperfect terminal IR sequences, and our results show that this arrangement is also suboptimal for transposition. Like Mos1, Mboumar-9 has a preference for one of its IRs; in this case, the IRL allows more efficient transposition *in vitro*. However, the effect of the different IR sequences is less than that for Mos1, reflecting the smaller difference between the *Mboumar-9* IRR and IRL: 2 bases out of 32, as compared with 4 bases out of 28 for Mos1. As with Mos1, the base at the 3′ transposon end is important for the transposition efficiency; replacing the 3′-guanine of the *Mboumar-9* IRL with adenine enhanced *Mboumar-*9 transposition almost 4-fold.

The favored model for Mos1 transposition invokes initial asymmetric binding of a transposase dimer to one transposon IR only, in a single-end complex (SEC2). Subsequent capture of a second, transposase-free end to form a PEC (37) promotes strand cleavage (38, 39). The differential affinity of the Mos1 transposase for the right and left IR sequences of its natural transposon would be expected to promote ordered PEC formation over a broad range of transposase concentrations, by limiting the frequency at which an active transposase dimer is bound at both ends at once. Consistent with this, *in vitro* PEC assembly was inhibited when the transposase concentration was in excess of that of the Mos1 ends (40). It remains to be seen whether there are additional differences in transposase interactions with IRR and IRL DNA within a nucleoprotein complex at an earlier stage of transposition before DNA cleavage and pairing of the ends, for example within the SEC2.

## Footnotes

2 The abbreviations used are:

IR: inverted repeat
IRR: right IR
IRL: left IR
PEC: paired-end complex
HTH: helix-turn-helix
TS: transferred strand
NTS: non-transferred strand
nt: nucleotide(s).
